# Anti-inflammatory, analgesic, and antipyretic potential of *Oxystelma esculentum* (L. f.) Sm. using *in vitro*, *in vivo*, and *in silico* studies

**DOI:** 10.3389/fphar.2023.1326968

**Published:** 2024-01-16

**Authors:** Asmaa E. Sherif, Muhammad Sajid-ur-Rehman, Muhammad Asif, Iram Qadeer, Kashif ur Rehman Khan

**Affiliations:** ^1^ Department of Pharmacognosy, College of Pharmacy, Prince Sattam Bin Abdulaziz University, Al-Kharj, Saudi Arabia; ^2^ Department of Pharmacognosy, Faculty of Pharmacy, Mansoura University, Mansoura, Egypt; ^3^ Department of Pharmacognosy, Faculty of Pharmacy, The Islamia University of Bahawalpur, Bahawalpur, Pakistan; ^4^ Department of Pharmacology, Faculty of Pharmacy, The Islamia University of Bahawalpur, Bahawalpur, Pakistan; ^5^ Department of Zoology, Government Sadiq College Women University, Bahawalpur, Pakistan; ^6^ Department of Pharmaceutical Chemistry, Faculty of Pharmacy, The Islamia University of Bahawalpur, Bahawalpur, Pakistan

**Keywords:** antipyretic, high-performance liquid chromatography, inflammation, *in silico* studies, *Oxystelma esculentum*

## Abstract

The objective of the current study was to evaluate the anti-inflammatory, analgesic, and antipyretic potential of *Oxystelma esculentum* using different animal models. The phytochemical profile was determined by assessing its total phenolic content (TPC) and total flavonoid content (TFC), followed by the high-performance liquid chromatography (HPLC) technique. The *in vitro* anti-inflammatory potential of *O. esculentum* ethanolic extract (OEE) was evaluated by lipoxygenase enzyme inhibition activity and a human red blood cell (HRBC) membrane stability assay. The *in vivo* anti-inflammatory potential of the plant was determined by the carrageenan-induced paw edema test, and the analgesic potential by the hot plate test, tail-flick test, formalin-induced analgesia, acetic acid-induced writhing activities, and yeast-induced elevation of body temperature. The values of total phenolic content (212.6 ± 3.18 µg GAE/g) and total flavonoid content (37.6 ± 1.76 µg QE/g) were observed. The results showed that OEE exhibited significant antioxidant capacity in DPPH (2,2-diphenyl-1-picrylhydrazyl) (266.3 ± 7.35 μmol TE/g), ABTS (2,2′-azino-bis(3-ethylbenzothiazoline-6-sulfonic acid) (1,066.3 ± 7.53 μmol TE/g), and FRAP (ferric reducing antioxidant power) (483.6 ± 3.84 μmol TE/g) assays. The HPLC analysis demonstrated phytocompounds with anti-inflammatory potential, such as chlorogenic acid, gallic acid, 4-hydroxybenzoic acid, caffeic acid, ferulic acid, and coumarin. The plant showed *in vitro* anti-inflammatory activity through the inhibition of lipoxygenase enzyme with a high percentage (56.66%) and HRBC membrane stability (67.29%). In *in vivo* studies, OEE exhibited significant (*p* < 0.05) anti-inflammatory (carrageenan-induced paw edema model), analgesic (hot plate test, tail-flick test, formalin-induced analgesia, and acetic acid-induced writhing), and antipyretic (rectal temperature reduction) responses at different doses (100, 300, and 500 mg/kg). Molecular docking studies showed significant binding affinities of phytocompounds compared to indomethacin and predicted various binding interactions for stable conformations. The results of *in vitro*, *in vivo*, and *in silico* studies supported the anti-inflammatory, analgesic, and antipyretic potential of *O. esculentum*.

## Graphical Abstract

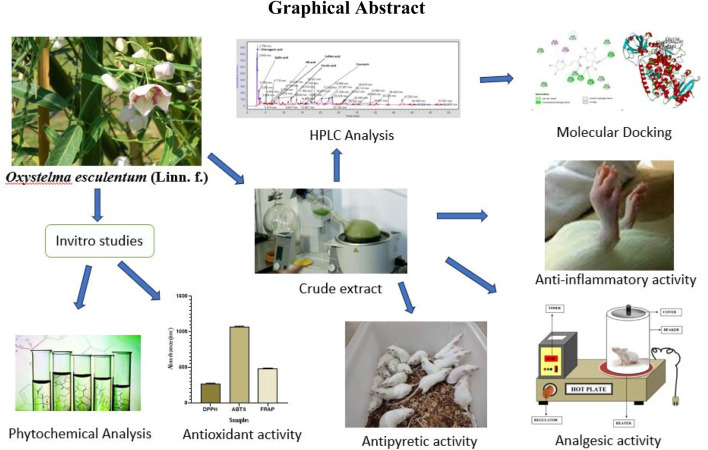

## 1 Introduction

Inflammation is a natural and essential biological response of the body to harmful stimuli, such as pathogens, damaged cells, or irritants. The purpose of inflammation is to eliminate the cause of cell injury, clear out damaged cells and tissues, and initiate tissue repair. It is a complex process involving the immune system, blood vessels, and various signaling molecules (Khalua et al., 2019). Inflammation may be acute or chronic, depending on the severity of the infection. Acute inflammation has been characterized as a vascular and cellular event (Zhang et al., 2020). Chronic inflammation may lead to the release of various biologically active substances by activated macrophages, resulting in tissue destruction, particularly in rheumatoid arthritis (RA) and hematological diseases (Figus et al., 2021).

Oxidative stress and inflammation have likewise been involved in the pathogenesis of acute and chronic damage to vital organs (Abdelghffar et al., 2022), also playing a significant role in the molecular pathogenesis of RA. In chronically affected joints, the normally thin synovium proliferates, thickens, and develops many villous folds. The synovial lining cells produce various materials, including collagenase and stromelysin, which contribute to cartilage destruction; interleukin-1 (IL-1) and TNF-alpha, which stimulate cartilage destruction, osteoclast-mediated bone absorption, and synovial inflammation; and prostaglandins, which potentiate inflammation (McInnes and Schett, 2011; Sokolove et al., 2012).

Arthritis is a condition of inflammation of joints that can make them stiff, painful, and difficult to move. Immunosuppressors (e.g., sulfasalazine, methotrexate, and hydroxychloroquine) are frequently prescribed for the treatment of some autoimmune chronic inflammatory diseases. Additionally, non-steroidal anti-inflammatory drugs (NSAIDs), which inhibit cyclooxygenase-1 (COX-1) and COX-2, are frequently used in chronic inflammatory diseases (Allawadhi et al., 2022). However, NSAIDs have serious side effects or cause other health problems, such as heart disease, diabetes, and cancer.

It is still unclear how to prevent long-term joint damage and functional decline, making it urgent to develop therapeutic drugs that are effective, affordable, and have a low risk of side effects for RA treatment (Wang et al., 2016). Natural products continue to be a source of therapeutic agents and have demonstrated useful applications (Muhamad Fadzil et al., 2021). The flora of the Cholistan Desert of Pakistan is rich in traditional plants that are used in the treatment of joint pain, inflammation, and arthritis (Rehman et al., 2015). Many plants in the Cholistan Desert are used for pain and inflammatory diseases, such as *Calotropis procera*, *Amaranthus viridis*, *Heliotropium crispum*, *Capparis decidua*, *Achyranthes aspera*, *Indigofera argentea*, *Ziziphus nummularia*, *Crotalaria burhia*, and *Withania somnifera* (Wariss et al., 2014).

One of the striking edible halophytes named *O. esculentum* (Linn. f.) R. Brown (Asclepiadaceae) has a wide range of distribution from Africa to Asia (Chishti et al., 2021). It is widely spread in the Balochistan plains, the Indus plains, and the Cholistan Desert of Pakistan (Khan and Qaiser, 2006). Fruits and leaves of this plant are used for treating burning urination and as diuretics. Decoction and infusion of the plant are used to treat sore throats, mouth ulcers, chronic fever, dysuria, and gonorrhea, and as hepatoprotective agents and blood purifiers. The methanol extract of *O. esculentum* exhibited strong anticancer, diuretic, and protective properties against paracetamol-induced hepatotoxicity (Ahmad et al., 2014; Wariss et al., 2014; Devi Maliga and Yogananth, 2016; Panda, 2019; Chishti et al., 2021). However, further work is required to evaluate the anti-inflammatory and antinociceptive abilities of *O*. *esculentum*. So this research work aims to validate its traditional uses as an anti-inflammatory, analgesic, and antipyretic agent. The novelty of this project is to explore the new source of the Cholistan Desert for its inhibitory effect on inflammatory enzymes and possible mechanisms of action in the treatment of inflammatory disorders.

## 2 Materials and methods

### 2.1 Plant material

The whole plant was collected from the desert area around Islamia University, Bahawalpur, Pakistan. The plant specimen was identified and authenticated by a plant taxonomist at the Cholistan Institute of Desert Studies, The Islamia University, Bahawalpur, with voucher number CIDS/IUB-0216/18 for further reference. The whole plant was shade-dried, ground, and placed in a glass container. Analytical-grade chemicals were used, including formalin (Riedel-de Haen, Germany), carrageenan and acetic acid (Sigma-Aldrich, United States), acetonitrile (HPLC-grade), indomethacin, and paracetamol from SAMI Pharmaceuticals. The standards of chlorogenic acids, 4-hydroxybenzoic acid, ellagic acid, gallic acid, catechin, ferulic acid, salicylic acid, coumarin, cinnamic acid, caffeic acid, and quercetin were purchased from Sigma-Aldrich, United States. Different doses of OEE (100, 300, and 500 mg/kg) were prepared in normal saline solution, while indomethacin 15 mg/kg and paracetamol 50 mg/kg were prepared in double-distilled water for administration.

### 2.2 Crude plant extract preparation

The pulverized plant was extracted using the Soxhlet extraction method in 500 mL of ethanol for 12 h before being concentrated in a rotary evaporator under reduced pressure. The extract was kept in the refrigerator at a temperature range of 4ºC–20°C in an airtight container until further use (Oikeh et al., 2020). The crude extract of *O. esculentum* was named OEE and subjected to preliminary phytochemical analysis.

### 2.3 Phytochemical screening

#### 2.3.1 Total phenolic content

The total phenolic content (TPC) of OEE was estimated using the Folin–Ciocalteu (FC) reagent method with slight modifications (Hayat et al., 2020; Younis et al., 2023). To estimate TPC, 200 μL of OEE was mixed with 1 mL of FC reagent (10% with deionized water), incubated for 20 min in the dark, and mixed with 800 μL of Na_2_CO_3_ (7.5%). The mixture was stirred and incubated again for 3 h, and then absorbance at 765 nm was measured using a UV spectrophotometer. Gallic acid was used as the standard, and the results were expressed as µg gallic acid equivalents/mg dry matter.

#### 2.3.2 Total flavonoid content

The total flavonoid content (TFC) of OEE was determined according to the already reported method (Hayat et al., 2020; Younis et al., 2023), with some modifications. To estimate TFC, 500 µL of the plant extract was added to 1,500 µL of (95%) methanol, 100 µL of AlCl_3_ (10%), 100 µL of sodium acetate, and 2.8 mL of double-distilled water. The stirred mixture was incubated at room temperature, and absorbance at 415 nm was measured. Quercetin was used as the standard, and the results were expressed as µg quercetin equivalents/mg dry matter.

### 2.4 Determination of antioxidant activities

#### 2.4.1 DPPH assay

The estimation of antioxidant activity by the DPPH assay was carried out using the method described in Adouni et al. (2022), with slight modifications. In brief, 50 μL of the plant extract was mixed with 2 mL of DPPH solution (89.7 μmol/L) in methanol. The mixed solution was incubated in the dark for 45 min, and absorption at 517 nm was measured. The DPPH radical scavenging activity was calculated from the calibration curve, and the values were expressed as μmol Trolox equivalents per gram of the dry extract (μmol TE/g).

#### 2.4.2 ABTS assay

The estimation of free radical scavenging activity by the ABTS assay was carried out using the method described in Adouni et al. (2022), with some modifications. In brief, an ABTS solution was prepared by mixing 50 mL of ABTS (2 mmol/L PBS) in 200 μL of K_2_S_2_O_8_ and incubated in the dark for 15 h. A measure of 3 μL of the plant extract was added to 300 μL of ABTS solution and again incubated for 30 min. The absorbance at 734 nm was measured, and the measurement was taken in triplicate. The ABTS scavenging activity was calculated from the calibration curve, and the values were expressed as μmol Trolox equivalents per gram of the dry extract (μmol TE/g).

#### 2.4.3 FRAP assay

The ferric-reducing antioxidant power (FRAP) assay was carried out by the method described by Jo et al. (2021) and Adouni et al. (2022), with some modifications. In brief, 50 μL of the plant extract was mixed with 150 μL of double-distilled water and 1,500 μL of FRAP reagent. The mixture was stirred and incubated in the dark for 2 h at 37°C. The absorbance at 593 nm was measured, and the ferric reducing power was calculated from the calibration curve. The results were expressed as μmol Trolox equivalents per gram of the dry extract (μmol TE/g).

### 2.5 HPLC analysis

For the HPLC analysis, the plant sample solution was prepared according to a previously reported method (Javed et al., 2020). OEE (50 mg) was mixed with methanol (20 μg/mL), distilled water (16 mL), and 6 M HCl (10 mL). The mixture was incubated at 25°C for 2 h and then filtered using a 0.45-µm nylon filter.

The mobile phase consists of a gradient elution using solvent A (1% formic acid in water) and solvent B (acetonitrile) at a flow rate of 1 mL/min and an injection volume of 0.02 mL of samples and standards. Gradient HPLC (Agilent, CA, United States) with the SIL-20A autosampler and a C-18 column (4.6 × 100 mm; 5 μm) was used with a VWD detector (284 nm). Data acquisition, peak integration, and calibrations were performed using Chromeleon software version 6.80 RS10. Identification of phenolic compounds in OEE was based on the comparison of the spectra and retention times of the samples against external standards. The quantification was carried out by external standardization using analytical curves, the limit of detection (LOD), and the limit of quantification (LOQ) (Abdelkhalek et al., 2020).

### 2.6 Experimental animals

Wistar albino rats (weighing 160–250 g) and albino mice (weighing 25–30 g) were used in *in vivo* studies. Before starting the experiments, the animals were housed for 1 week in a 12-h light and 12-h dark environment at 25 ± 2 °C and 50%–55% humidity and fed with standard feed and water. All experimental animal procedures were approved by the Pharmacy Animal Ethics Committee (approval number PAEC 20/30), Faculty of Pharmacy, The Islamia University of Bahawalpur, Punjab, Pakistan.

### 2.7 Acute toxicity study

An acute toxicity study was performed on albino mice (weighing 25–30 g), which were divided into five groups (n = 5). After overnight fasting, OEE at different doses (0.5, 1, 3, and 5 g/kg b.w./day) was provided to the animals by gastric intubation. The animals were then observed closely for 48 h for 14 days. Animal behaviors such as general activity, touch response, irritability, tail grip response, body tone, tremor, convulsion, lacrimation, urination, defecation, breathing, and writhing reflex, along with loss of weight and death, were also observed (Brígido et al., 2021).

### 2.8 Anti-inflammatory assays

#### 2.8.1 *In vitro* lipoxygenase inhibitory activity

The lipoxygenase (LPO) inhibition potential of OEE was determined according to the already reported method (Alshawush et al., 2022) using an enzyme assay kit (cat no. ab241038, Abcam, United States). A measure of 10 μL of serial concentrations of the sample (25 μg/mL) was mixed with a reaction mixture containing buffer solution (0.2 M, pH = 9) and 20 µL of lipoxygenase solution and incubated for 5 min at 25 °C. Linoleic acid solution (10 µL) as a substrate was added to initiate the reaction, and the mixture was incubated for 10 min at room temperature. Finally, % LPO inhibition was calculated according to the following formula:

$$\%\;LPO\;inhibition=\frac{A-B}{A}\times 100,$$

where A = (absorbance of control–absorbance of background) is the activity of the enzyme without the sample and B = (absorbance of sample–absorbance of background) is the activity of the enzyme with the sample. IC_50_ values were determined using the equation of nonlinear regression with the help of Microsoft Office Excel. The experiments were performed in triplicate.

#### 2.8.2 *In vitro* anti-inflammatory assay

The RBC membrane stabilization assay was carried out using a healthy volunteer’s human blood sample. Alsever’s solution, an anti-coagulant, was added to the blood and then centrifuged for 15 min at 1,100 RCF. A mixture containing 1 mL phosphate buffer, 2 mL hyposaline (0.36%), 0.5 mL RBC suspension (10% in normal saline), and 0.5 mL of different concentrations of OEE (25, 50, 100, 200, 400, and 800 μg/mL) was prepared. A similar mixture of different concentrations of indomethacin was also prepared. The mixtures were incubated for 30 min at 37°C and then centrifuged at 1,100 RCF for 20 min. The absorbance of the supernatant at 560 nm was measured using a spectrophotometer (Sajid-Ur-Rehman et al., 2021). %Age of RBC membrane stability was calculated using the following formula:

$$\text{Protection }\%=100-\frac{\text{Optical}\;\text{density}\;\text{of}\;\text{test}\;\text{sample}}{\text{optical}\;\text{density}\;\text{of}\;\text{control}}\times 100.$$

The experimental protocol was approved by the Pharmacy Institutional Ethical Committee (PHEC).

#### 2.8.3 Carrageenan-induced paw edema

The carrageenan-induced paw edema model was used to evaluate the anti-inflammatory impact of OEE (Basit et al., 2023; Tabassum et al., 2023). A measure of 100 μL of freshly prepared 1% carrageenan was administered to the right hind paw of rats 1 h after treatment administration, except for the control group. The normal paw diameter of all animals was measured using a digimatic caliper (Mitutoyo, Japan). Before carrageenan administration, the rats were given vehicles (normal saline, 5 mL/kg, PO), indomethacin (standard drug, 15 mg/kg, i.p.), and different doses of the plant extract (100, 300, and 500 mg/kg, i.p.). The change in paw size (mm) was again measured after the induction of inflammation at 0 h up to 6 h. The following formula was used to calculate the percentage inhibition of paw edema:

$$Inhibition\;\%=\frac{Control\;mean-treated\;mean}{Control\;mean}\times 100.$$

### 2.9 Analgesic potential of OEE

The analgesic effect of OEE was measured by hot plate, tail immersion, formalin-induced licking, and writhing tests. Wistar albino rats (weighing 160–220 g) were divided into 6 groups of 6 animals each. Normal saline (5 mL/kg) was given to the control group; indomethacin (15 mg/kg) was given to the standard group; and treated groups were administered 100, 300, and 500 mg/kg doses of OEE.

#### 2.9.1 Hot-plate assay

The hot-plate assay was performed as described in Tabassum et al. (2023), with minor modifications. The reflexes of all the rats, such as paw licking, lifting, or jumping, were noted when placed on the hot plate (VELP^®^, Italy) at a fixed temperature of 50°C–55°C. The reaction time before and after the therapy at 30, 60, 90, and 120 min was recorded. To avoid paw injury, 25 s was set as the cut-off time.

#### 2.9.2 Tail-flick assay

The tail-flick test was carried out according to the previously reported method (Tabassum et al., 2023). The tail end of the rat (1–3 cm) was dipped in hot water at 50°C–55°C, and the flick reaction (time in seconds required to remove the tail from hot water) of all rats was calculated before and after administration of the testing drug and extract at 30, 60, 90, and 120 min. A latency interval of 25 s was set as the cut-off time to avoid tissue damage.

#### 2.9.3 Formalin nociceptive assay

The formalin nociceptive test is one of the best ways to analyze acute pain in rodents. There are two distinct licking/biting responses or periods following the injection, separated by a quiescent time. The formalin nociceptive test was performed according to the previously reported method (Sajid-Ur-Rehman et al., 2021) with slight modifications. A measure of 0.05 mL of formalin (2.5% mL) was injected into the paw tissues of all experimental rats except the control group. Half an hour after the injection, the rats were treated with different doses of OEE along with the standard drug. The rats showed licking and biting responses that were observed in the first phase (0–5 min) and the second phase (15–30 min) after formalin injection (López-Cano et al., 2017).

#### 2.9.4 Writhing test in mice

The analgesic potential of OEE was determined using an acetic acid-induced writhing test conducted according to Sajid-Ur-Rehman et al. (2021), with slight modifications. Thirty mice (20–30 g) were randomly divided into five groups (n = 6). Normal saline (5 mL/kg) was given to the control group, i.p., the standard group was given indomethacin 15 mg/kg, i.p., and the treated group received OEE at different doses (100, 300, and 500 mg/kg) orally. A measure of 0.1 mL of acetic acid (0.6% v/v) was injected i.p. 30 min after the administration of drugs. The total number of writhes (abdominal muscular contractions) was counted for 5–30 min after the administration of acetic acid.

### 2.10 Antipyretic activity

The antipyretic effect of OEE was evaluated with a fever induced by an aqueous suspension of Brewer’s yeast following the established standard method with slight modifications (Dutta et al., 2020). Thirty Wistar albino rats were divided randomly into five groups (n = 6) and kept fasting with the provision of water *ad libitum*. The normal body temperature of all animals was noted using a digital thermometer. Pyrexia (fever) was induced by injecting a 20% w/v aqueous Brewer’s yeast (10 mL/kg) suspension in the dorsum region of the rats. The animals were then fasted overnight, and the rectal temperature was recorded again. Animals that showed a temperature rise (+0.5°C) were included in the study (Sobeh et al., 2020). The animals with pyrexia were orally administered OEE (100, 300, and 500 mg/kg), and the standard drug (paracetamol, 50 mg/kg) and normal saline (5 mg/kg) were administered to the control group. The rectal temperature of each animal in each group was recorded periodically at the first, second, third, and sixth hours after the drug administration.

### 2.11 Molecular docking studies

#### 2.11.1 Preparation of enzyme and ligand molecules

Molecular docking analysis was carried out using different tools, such as Discovery Studio, Babel, AutoDock Vina, PyRx, and mgltools (Shahid et al., 2023). Ligand structures (3D) were downloaded from the PubChem database in SDF (structure-data file) format and optimized using Babel. High-resolution lipoxygenase enzyme (1N8Q) (Borbulevych et al., 2004) was downloaded from the RSCB Protein Data Bank (accessed on 15 May 2023), and before docking, it was prepared using Discovery Studio 2021 Client. All inhibitors and water molecules were removed.

#### 2.11.2 Docking interactions of phytochemicals

Ligands and prepared enzymes were uploaded to AutoDock Vina, embedded in PyRx, and docking was performed (Khan et al., 2023). It is a very useful tool in computer-aided drug development studies. First, the protein molecule (lipoxygenase) was downloaded from the Protein Data Bank (PDB) in PDB format. The PDB ID of lipoxygenase is 1YGE. The protein preparation process was conducted using Discovery Studio 2021 Client. All chains, except for the A chain, and any water molecules and ligands that were already attached to the active site were eliminated from the protein molecules. Subsequently, polar hydrogen molecules were added to the proteins, and the resulting structures were saved as a PDB file. Secondary metabolites chosen from the high-performance liquid chromatography (HPLC) analytical technique table and standard compounds were downloaded from the PubChem database in SDF format. Then, the prepared protein molecules were uploaded to PyRx software and subjected to automated docking, and macromolecule options were made. The ligands, on the other hand, were uploaded into PyRx from Open Babel to undergo the necessary preparation steps. Afterward, they were minimized and converted to the PDBQT format. Then, a grid box was formed with specific dimensions: (X: 105.5460), (Y: 67.6652), and (Z: 57.3730). Finally, the interactions were visualized using Discovery Studio.

### 2.12 Statistical analysis

The values are presented as the mean ± standard error of the mean (SEM). The treatment and standard groups were compared with their respective saline-treated controls using analysis of variance followed by Bonferroni *post hoc* tests. GraphPad Prism software (GraphPad, United States) was used to construct graphs and analyze the data. *p* < 0.05 was considered significant.

## 3 Results

### 3.1 Phytochemical analysis

The preliminary phytochemical profiling of OEE revealed the presence of bioactive components such as alkaloids, glycosides, flavonoids, phenols, tannins, saponins, and terpenoids (Table 1). These secondary metabolites have been found to have therapeutic or medicinal properties such as anti-inflammatory, anticancer, and antimicrobial effects (Zhao et al., 2021).

**TABLE 1:** Presence of secondary metabolites in OEE.

Sr. No	Secondary metabolites	Test name	OEE
1	Alkaloids	Dragendorff test	+
		Wagner’s test	+
2	Glycosides	Borntrager’s test	−
		Sodium hydroxide test	+
3	Flavonoids	Shinoda test	+
4	Phenols	Ferric chloride test	+
5	Tannins	Lead acetate test	+
6	Saponins	Frothing test	+
7	Terpenoids	Salkowski reaction test	+
9	Coumarins	Sodium hydroxide test	+

(+) = presence; (−) = absence.

### 3.2 Total phenolic and flavonoid contents

**FIGURE 1:**
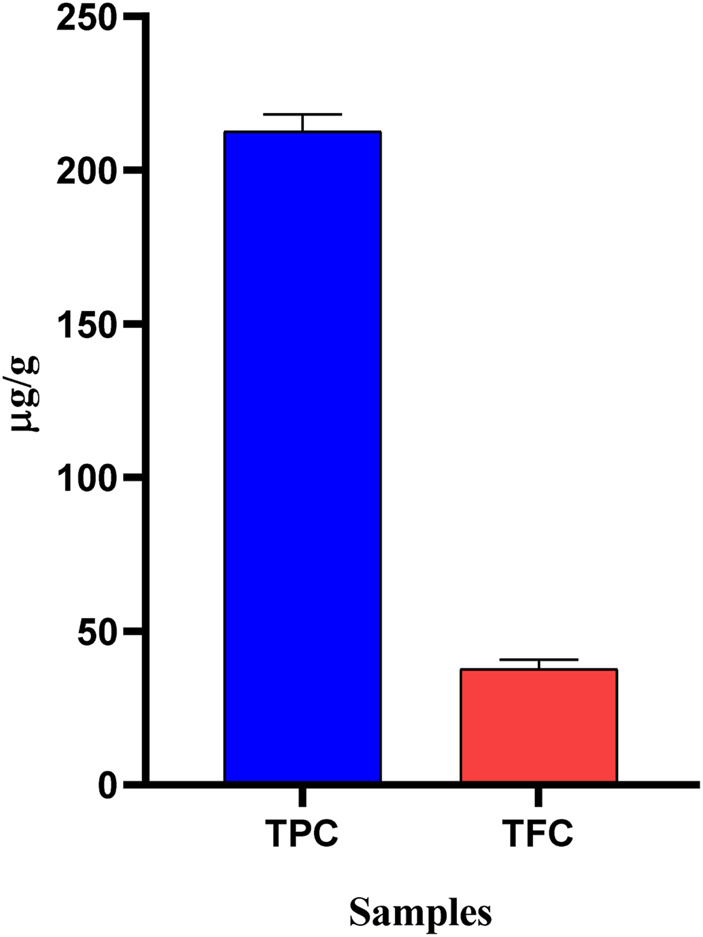
Total phenolic (µg GAE/g) and flavonoid (µg QE/g) contents of OEE. The values are expressed as mean ± SEM, n = 3.

Phenolic compounds and flavonoids naturally have cardio-protective, anticancer, anti-diabetic, anti-aging, and neuroprotective properties, and a plant enriched with them may boost the body’s antioxidant capacity (Patle et al., 2020). Therefore, it is crucial to identify these compounds in plant samples. OEE was found to possess a total phenolic content of 212.6 ± 3.18 (µg GAE/g) and a flavonoid content of 37.6 ± 1.76 (µg QE/g). The high contents of phenolics and flavonoids (Figure 1) suggested that OEE may contain antioxidant properties; hence, this plant could be a potential candidate for medicinal use in the treatment of various ailments.

### 3.3 Antioxidant activities

**FIGURE 2:**
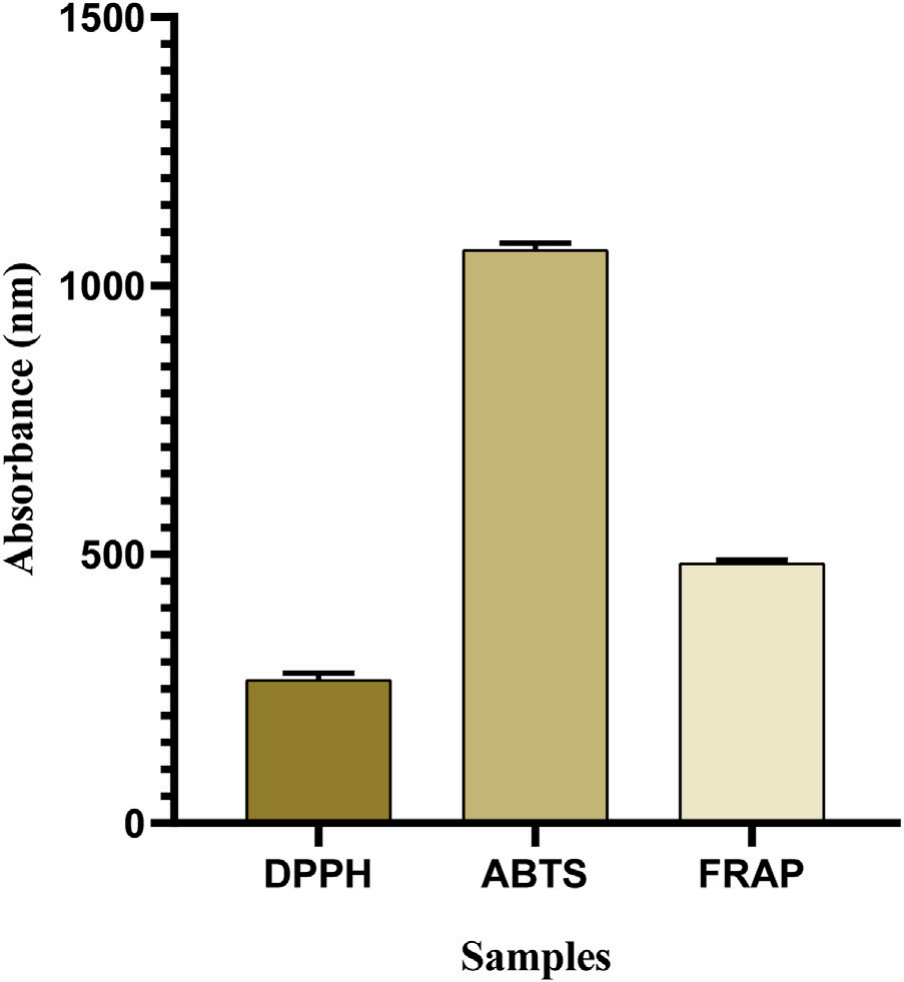
Antioxidant capacity of OEE evaluated using DPPH, ABTS, and FRAP assays is expressed in μmol TE/g. All resulting values are denoted as mean ± SEM. n = 3.

The DPPH assay is the most commonly used approach to determine whether phytocompounds can behave as hydrogen donors or free radical scavengers (Sheik et al., 2023). The results showed that OEE exhibited significant antioxidant potential in DPPH (266.3 ± 7.35 μmol TE/g), ABTS (1066.3 ± 7.53 μmol TE/g), and FRAP (483.6 ± 3.84 μmol TE/g) assays (Table 2; Figure 2).

**TABLE 2:** Representation of the antioxidant activity of OEE using DPPH, ABTS, and FRAP assays.

DPPH (μmol TE/g)	ABTS (μmol TE/g)	FRAP (μmol TE/g)
266.3 ± 7.35	1066.3 ± 7.53	483.6 ± 3.84

DPPH, ABTS, and FRAP are expressed in μmol TE/g. All values are denoted as mean ± SEM.

### 3.4 HPLC analysis

**FIGURE 3:**
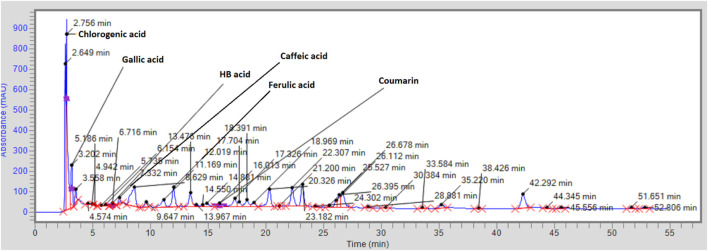
HPLC spectra for polyphenols.

The HPLC analysis of OEE supported the phytochemical screening result and showed the presence of phytocompounds with reported anti-inflammatory and analgesic potential (Figure 3; Table 3). 4-Hydroxybenzoic acid and chlorogenic acid were found in the highest concentrations, followed by gallic acid, ferulic acid, coumarin, and caffeic acid.

**TABLE 3:** Polyphenols (μg/g) identified using HPLC analysis.

Compound	RT (min)	Area	K-factor	Quantity (μg/g)
Chlorogenic acid	2.756	8,836,517	0.000013	114.8
Gallic acid	3.202	1,144,412.3	0.000088	100.7
HB acid (4-hydroxybenzoic acid)	6.154	862,431	0.00016	137.9
Caffeic acid	7.332	1,708,361	0.000059	90.5
Ferulic acid	12.019	1,303,525.2	0.0000767	99.9
Coumarin	16.013	83,212	0.0012	99.8

### 3.5 Acute toxicity studies

Acute toxicity testing at the oral limit dose of OEE caused no death in the tested animals. No lethal effect was noted during the short- and long-term observations. No toxicity signs or behavioral changes were observed in the animals throughout the 14-day study period; therefore, it was concluded that OEE is considered safe.

### 3.6 Anti-inflammatory assay

#### 3.6.1 Lipoxygenase inhibition assay

OEE’s effect as a lipoxygenase inhibitor indicated that the plant extract may be responsible for the conversion of polyunsaturated fatty acids into anti-inflammatory compounds. The IC_50_ value of lipoxygenase inhibitory activity (Table 4) was calculated as 8.2 μg/mL compared to that of the standard control (IC_50_ = 0.29 μg/mL).

**TABLE 4:** Representation of the lipoxygenase inhibition potential (IC_50_) of OEE.

Plant extract	% Inhibition ±SEM	IC_50_ (μg/mL)
OEE	56.66	8.2
Baicalein	96.27	0.29

#### 3.6.2 *In vitro* anti-inflammatory assay

**FIGURE 4:**
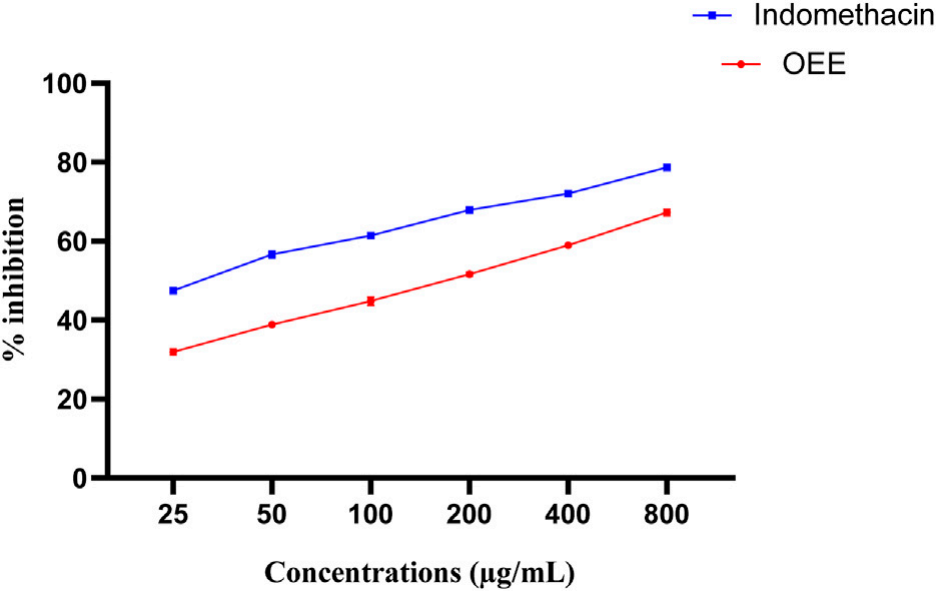
*In vitro* anti-inflammatory activity of OEE using a human red blood cell membrane stabilization model, representing the % inhibition of OEE and the standard drug (indomethacin). The results were evaluated at the end of the treatment by one-way ANOVA, followed by Bonferroni’s test. The result represents the mean ± SEM for n = 3.

The human red blood cell stabilizing assay against hyposaline-induced RBC lysis was observed. OEE significantly stabilized the RBCs from lysis (31.96%, 38.91%, 44.81%, 51.65%, 58.92%, and 67.29%) at various concentrations (25, 50, 100, 200, 400, and 800 μg/mL) compared to the standard drug (indomethacin) (Figure 4).

#### 3.6.3 Carrageenan-induced paw edema

**FIGURE 5:**
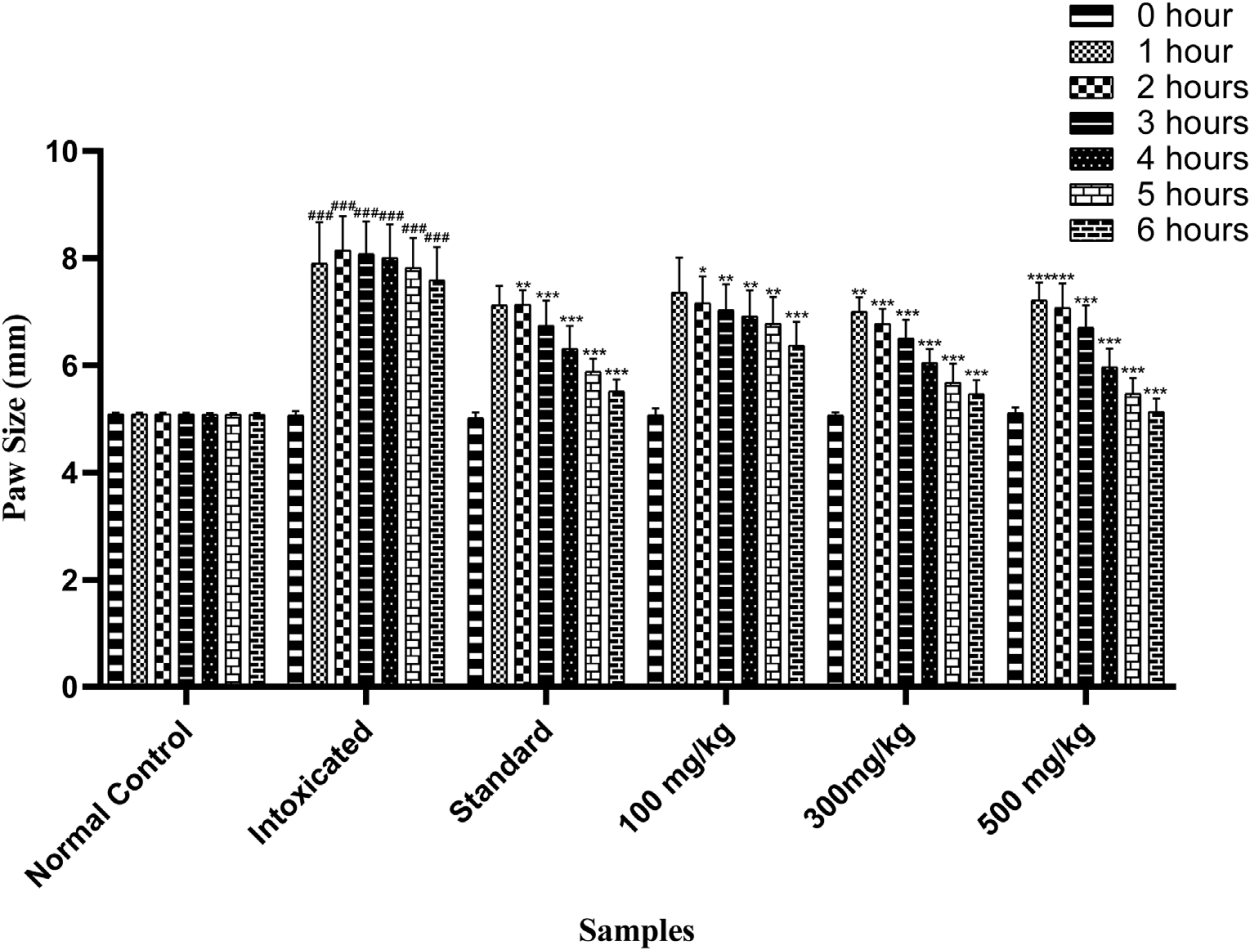
Effect of OEE on carrageenan-induced edema at different doses. The results were evaluated at the end of the treatment by one-way ANOVA, followed by Bonferroni’s test. Each point in the bar graph represents the mean ± SEM for n = 6 experiments on rats. *p* < 0.001 (***), *p* < 0.01 (**), and *p* < 0.05 (*) when comparing the treatment group to the intoxicated group.

OEE at different doses (100, 300, and 500 mg/kg) has shown anti-inflammatory effects against carrageenan-induced inflammation in animal studies. At 100 mg/kg, anti-inflammatory effects occurred after the third to sixth hour (7.03 ± 0.19, 6.91 ± 0.19, 6.78 ± 0.2, and 6.37 ± 0.18 mm) after inflammation. At 300 mg/kg, OEE showed an anti-inflammatory response from the second to sixth hour (6.77 ± 0.11, 6.50 ± 0.13, 6.05 ± 0.1, 5.67 ± 0.14, and 5.46 ± 0.1 mm), whereas a 500 mg/kg dose showed significant anti-inflammatory effects from the second to sixth hour (7.07 ± 0.18, 6.7 ± 0.16, 5.97 ± 0.13, 5.47 ± 0.11, and 5.13 ± 0.09 mm) when compared to the intoxicated group (Table 5; Figure 5).

**TABLE 5:** Effect of OEE on carrageenan-induced edema.

Sr. No.	0 h	1 h	2 h	3 h	4 h	5 h	6 h
Normal control	5.09 ± 0.01	5.09 ± 0.01	5.09 ± 0.01	5.08 ± 0.01	5.08 ± 0.01	5.08 ± 0.01	5.08 ± 0.01
Intoxicated	5.06 ± 0.03	7.9 ± 0.31	8.14 ± 0.26	8.08 ± 0.24	8.0 ± 0.25	7.82 ± 0.22	7.58 ± 0.25
Standard (indomethacin)	5.01 ± 0.04	7.12 ± 0.14	7.13 ± 0.1	6.74 ± 0.18	6.31 ± 0.17	5.88 ± 0.09	5.51 ± 0.08
100 mg/kg	5.07 ± 0.05	7.35 ± 0.26	7.16 ± 0.2	7.03 ± 0.19	6.91 ± 0.19	6.78 ± 0.2	6.37 ± 0.18
300 mg/kg	5.06 ± 0.02	7.0 ± 0.11	6.77 ± 0.11	6.50 ± 0.13	6.05 ± 0.1	5.67 ± 0.14	5.46 ± 0.1
500 mg/kg	5.1 ± 0.04	7.21 ± 0.13	7.07 ± 0.18	6.7 ± 0.16	5.97 ± 0.13	5.47 ± 0.11	5.13 ± 0.09

The results are expressed as mean ± SEM, n = 6.

### 3.7 Analgesic activity

#### 3.7.1 Hot-plate method

**FIGURE 6:**
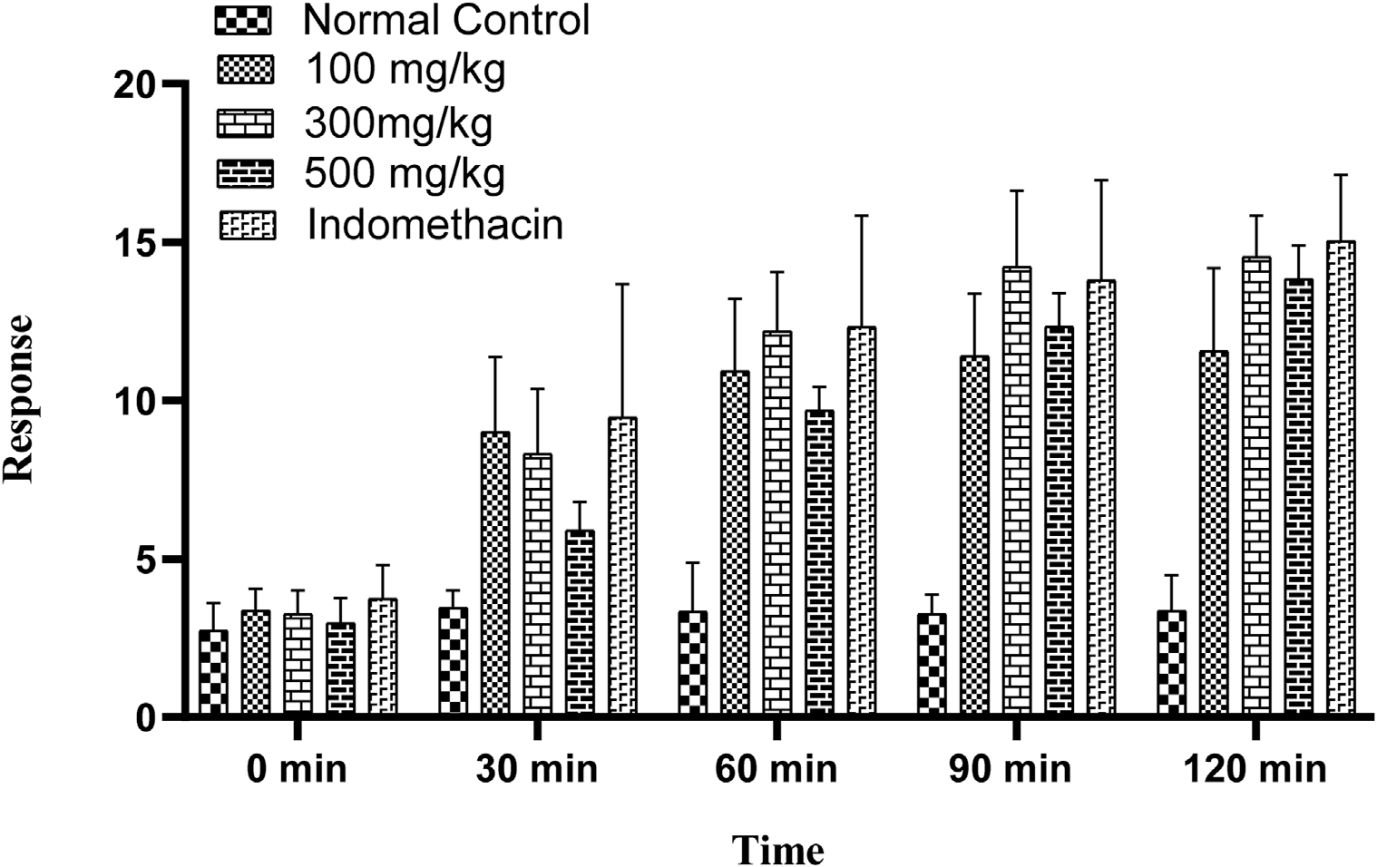
Hot-plate sensitivity test showing an increase in latency time with different doses of OEE and the indomethacin drug as the standard.

OEE showed significant heat sensitivity response in the hot-plate method at various time intervals (30, 60, 90, and 120 min) at different doses (100, 300, and 500 mg/kg). The standard drug indomethacin also significantly reduced pain at all given time intervals (Figure 6).

#### 3.7.2 Tail-flick method

**FIGURE 7:**
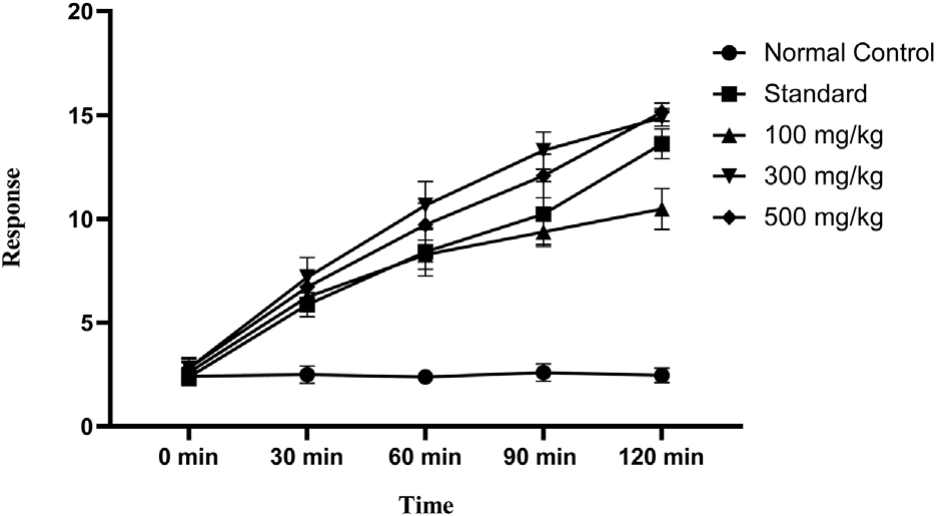
In the tail immersion test, there was an increase in delay time in thermal stimuli with various doses of OEE and the indomethacin drug as the standard.

The tail-flick latency to thermal stimuli was evaluated using a hot water tail immersion test, and all doses of OEE (100, 300, and 500 mg/kg) exhibited significant results in the delay of the response to thermal analgesia when compared to the control group (Figure 7).

#### 3.7.3 Formalin-induced pain test

**FIGURE 8:**
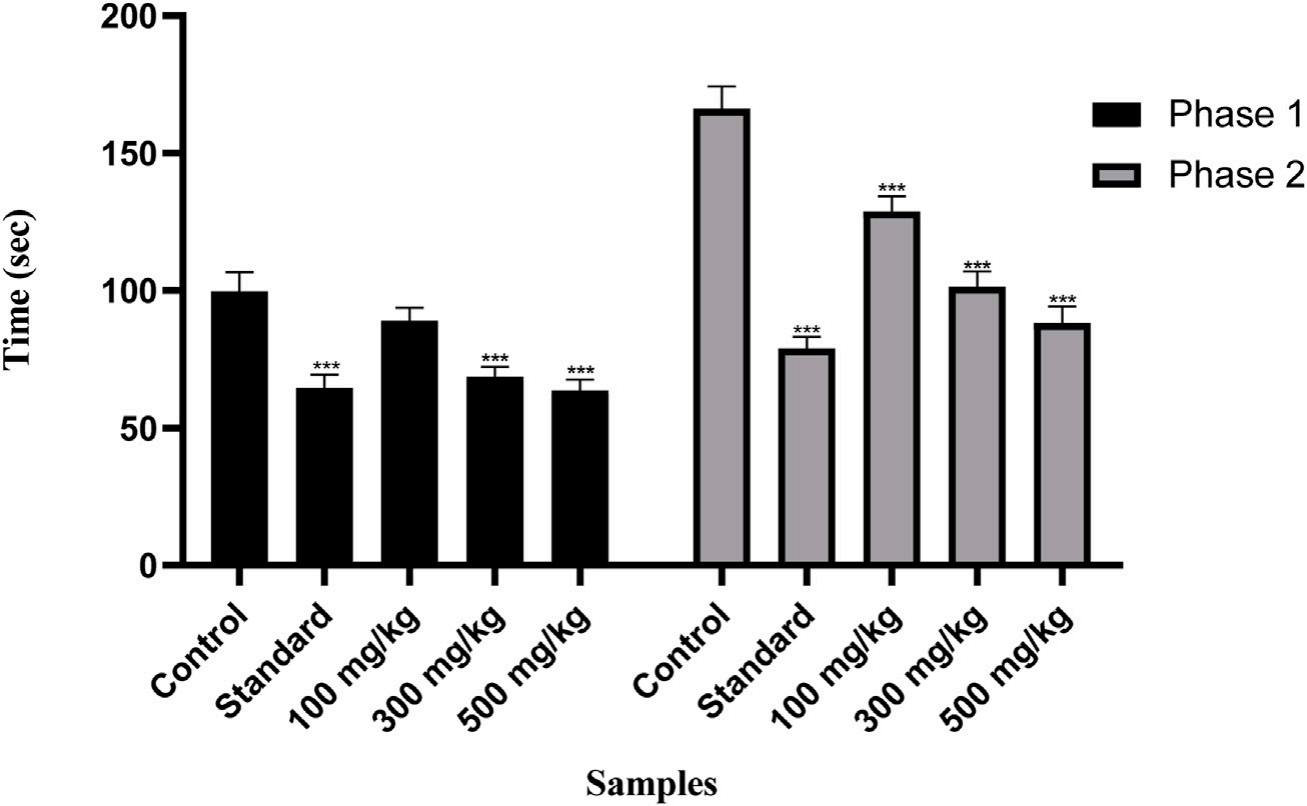
There was a decrease in pain response in the formalin-induced pain test with OEE and the indomethacin drug. The results were evaluated at the end of the treatment by one-way ANOVA, followed by Bonferroni’s test. Each point in the bar graph represents the mean ± SEM for n = 6 experiments on rats. *p* < 0.001 (***), *p* < 0.01 (**), and *p* < 0.05 (*) when comparing the treatment group to the intoxicated group.

Figure 8 shows the remarkable potential of OEE against formalin-induced pain. OEE at doses of 100, 300, and 500 mg/kg presented dose-related reductions in the first neurogenic phase (10.8%, 31.2%, and 36.2%) and second inflammatory phases (37.3%, 38.9%, and 46.9%) of the formalin test. Indomethacin showed significant inhibition in neurogenic/inflammatory pain in both the early (35.2%) and late (52.4%) phases.

#### 3.7.4 Writhing test in mice

**FIGURE 9:**
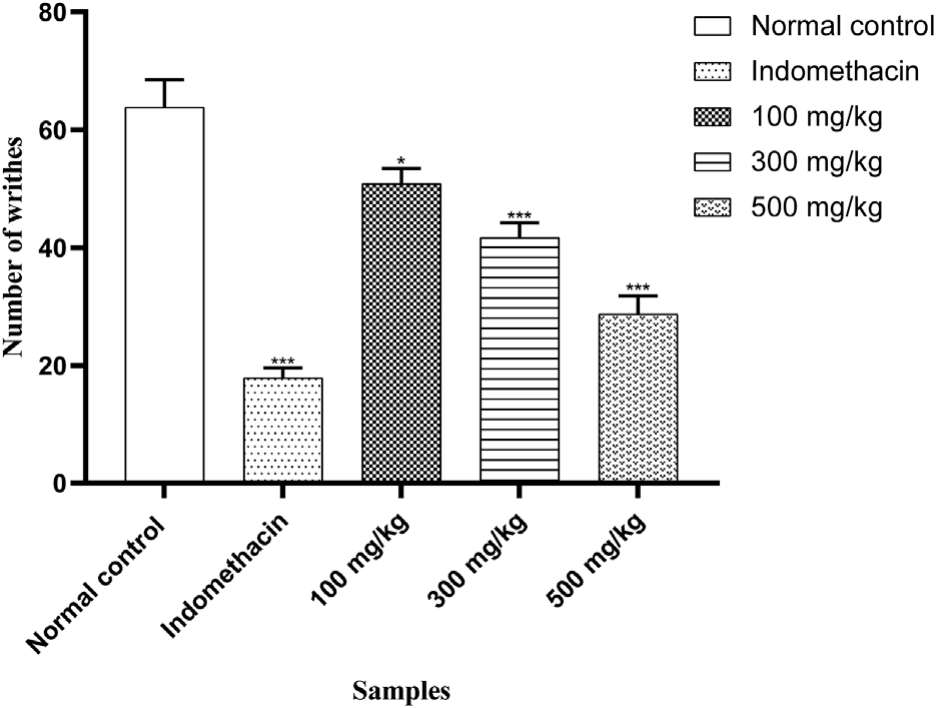
There was a decrease in abdominal contractions (writhing response) in the acetic acid-induced writhing test with OEE and the indomethacin drug. The results were evaluated at the end of the treatment by one-way ANOVA, followed by Bonferroni’s test. Each point in the bar graph represents the mean ± SEM for n = 6 experiments on rats. *p* < 0.001 (***), *p* < 0.01 (**), and *p* < 0.05 (*) when comparing the treatment group to the intoxicated group.

OEE (100, 300, and 500 mg/kg) showed antinociceptive effects in the acetic acid-induced writhing model in a dose-dependent manner. OEE significantly inhibited the number of abdominal contractions (writhes) (*p* < 0.05) when compared with the untreated (control) and standard groups (Figure 9).

### 3.8 Antipyretic activity

**FIGURE 10:**
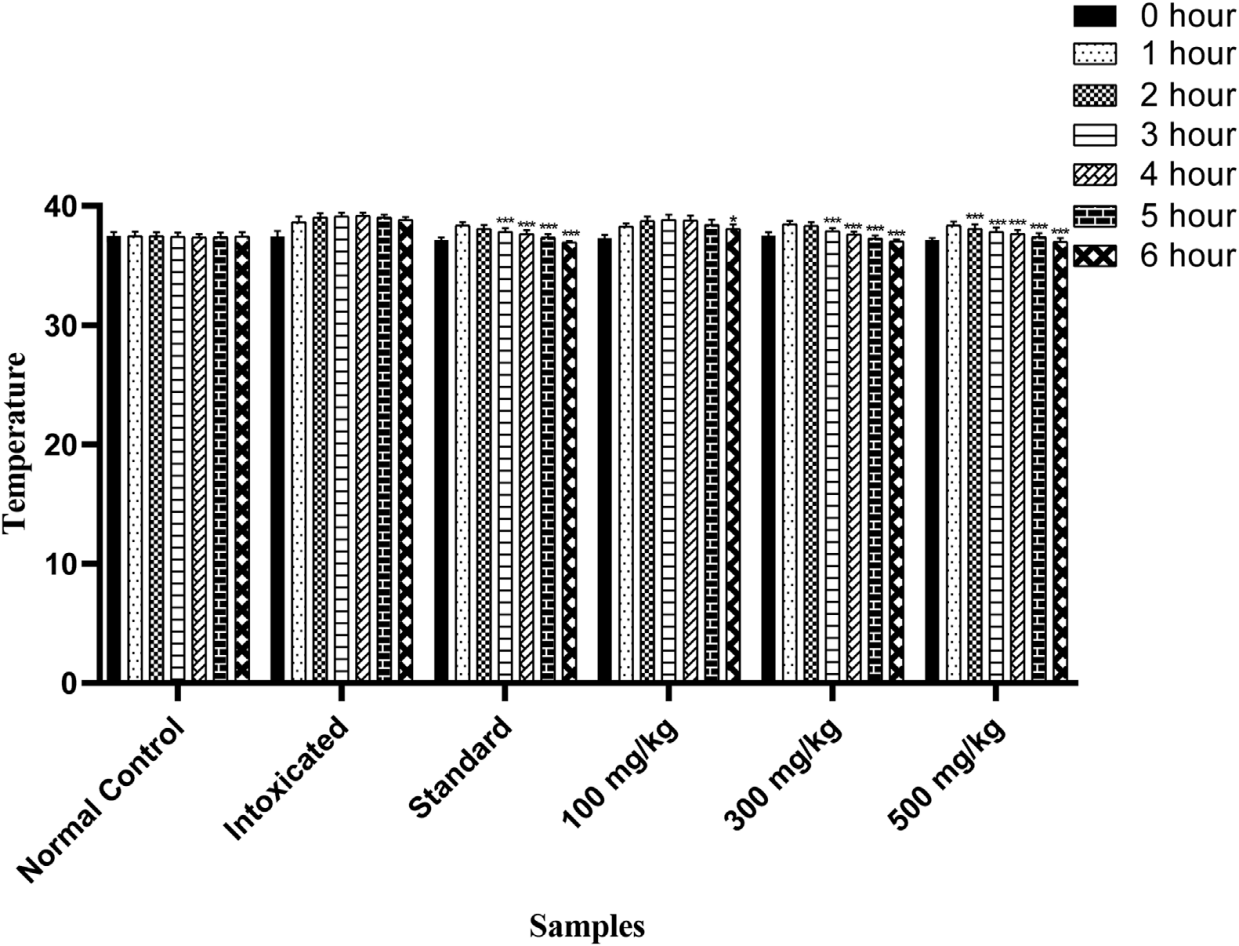
Antipyretic activity of OEE was observed in falling rectal temperature in yeast-induced pyrexia at different doses of OEE and paracetamol drug. The results were evaluated at the end of the treatment by one-way ANOVA, followed by Bonferroni’s test. Each point in the bar graph represents the mean ± SEM for n = 6 experiments on rats. *p* < 0.001 (***), *p* < 0.01 (**), and *p* < 0.05 (*) when comparing the treatment group to the intoxicated group.

The antipyretic potential of OEE was estimated by observing the progressive reduction in the rectal temperature in all treated rats. The standard drug (paracetamol) showed a significant reduction in temperature from the third to sixth hour, while OEE significantly reduced the temperature from the third to sixth hour (300 mg/kg of the plant extract) and from the second to sixth hour post-treatment at the dose of 500 mg/kg (*p* < 0.05) (Figure 10).

### 3.9 Molecular docking results

To better understand the inhibition potential of understudied phenolic compounds and to compare these data with enzyme inhibition findings, six phenolic compounds identified using the high-performance liquid chromatography analytical technique and one standard compound were docked against lipoxygenase proteins. Chlorogenic acid showed the highest binding affinity (−8.8 K cal/mol) among all phytocompounds reported by the HPLC analysis, while indomethacin (standard) presented the maximum binding affinity (−9.1). The binding affinity of other phenolic compounds is presented in Table 6. In addition to van der Waals forces, various interactions showed the binding mechanism of chlorogenic acid with the enzyme active site. Conventional hydrogen bonding was observed between the carbonyl moiety of caffeic acid and the Glu-197 amino acid residue. Residues Tyr-197 and Arg-240 also interacted with the carbonyl moiety of quinic acid through hydrogen bonding, while Glu-194 interacted with the hydroxyl group at the C-1 position through hydrogen bonding. A carbon–hydrogen bond was also observed between Asp-255 and oxygen at the C-4 position of the quinic acid ring of the chlorogenic acid molecule. The pi bonds of the aromatic ring were linked to Ala-263 and Leu-258 residues through the pi–alkyl interaction. This interaction contributed to the stable conformation of chlorogenic acid and the active site residues of the enzyme. A prominent binding interaction of gallic acid with Asn-186, Lys-156, Ser-157, and Arg-200 amino acid residues of the enzyme, hydroxyl groups at C-3, -4, and -5 positions, and carbonyl groups through conventional hydrogen bonding was observed.

**TABLE 6:** Binding affinity of all reported phenolic compounds and standard drugs.

Compound name	PubChem ID	Binding affinity
Chlorogenic acid	1794427	−8.8
Gallic acid	370	−6.9
4-Hydroxybenzoic acid	135	−6.3
Caffeic acid	689043	−6.8
Ferulic acid	445858	−6.7
Coumarin	323	−6.4
Indomethacin	3715	−9.1

Hydroxybenzoic acid also showed conventional hydrogen bonding with Val-144 and Phe-162 through the linkage of the hydroxyl of the carboxylic group. The pi–sigma and pi–pi T-shaped interactions were observed with Val-539 and Phe-161, respectively. At the same time, some van der Waals interactions may also contribute to the stable conformation of the hydroxybenzoic acid and enzyme complex. Two amino acid residues of the enzyme active site, Val-144, and Lys-545, interacted with carbonyl and hydroxyl groups of caffeic acid at the C-9 position of the molecule, respectively, while an unfavorable acceptor–acceptor interaction was observed between the hydroxyl group at C-9 and the Ala-163 residue of the enzyme. Moreover, a pi–alkyl interaction between Lys-545 and the aromatic ring of the ligand driven by the van der Waals forces was also observed.

**FIGURE 11:**
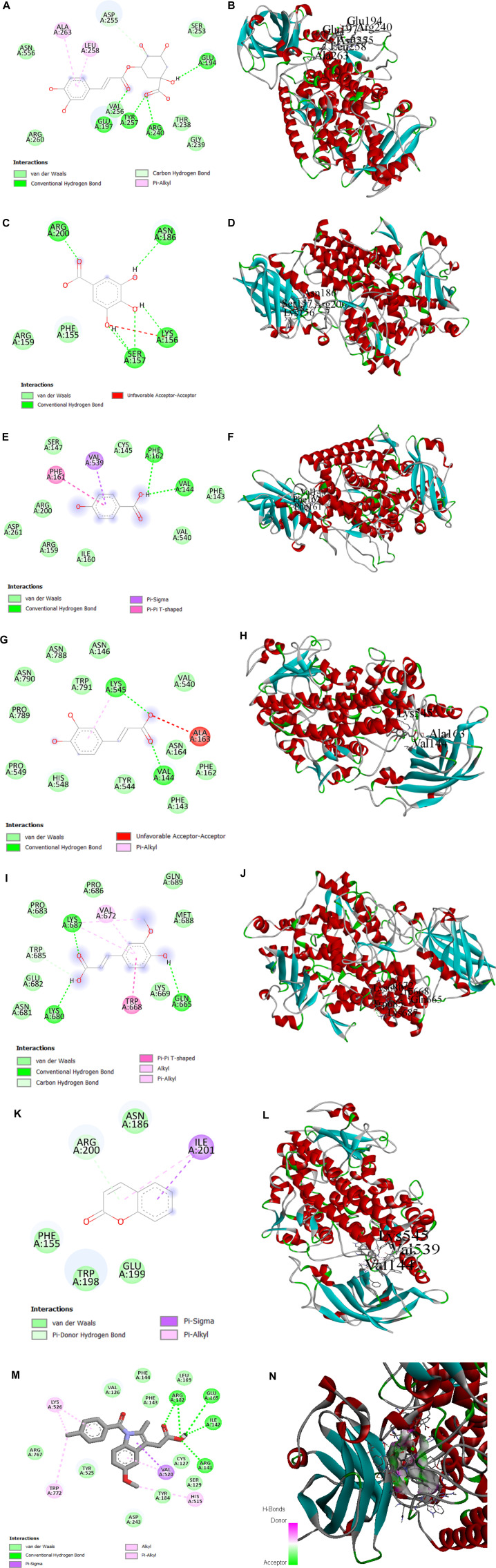
**(A, B)** 2D and 3D representations of binding interactions of chlorogenic acid, **(C, D)** gallic acid, **(E, F)** HB acid (4-hydroxybenzoic acid), **(G, H)** ferulic acid, **(I, J)** caffeic acid, and **(K, L)** coumarin (the ligand with the highest binding score) with lipoxygenase active site residues. **(M, N)** 2D and 3D representations of binding interactions of indomethacin (standard).

Ferulic acid also showed interactions of the carbonyl and hydroxyl groups at C-9 and the hydroxyl group at C-4 with Lys-680, Lys-687, and Gln-665 amino acid residues through hydrogen bonding. The carbon–hydrogen bond was observed between –OH at C-9 and the Trp-685 amino acid residue, while the pi interactions were depicted by three residues (Trp-668, Val-672, and Lys-687) with the aromatic ring of the molecule. Coumarin showed a pi–donor hydrogen bond between the Arg-200 residue and the pi bond at the C-3 position of the coumarin ring system. Moreover, Ile-201 also showed the pi–alkyl interaction at the position. The pi–sigma interaction was observed between Ile-201 and the aromatic ring system. Collectively, the interactions showed stable conformations for the enzyme–ligand complexes involving multiple binding forces (Figure 11).

## 4 Discussion

In this study, *O. esculentum* was evaluated for its chemical profiling and anti-inflammatory, analgesic, and antipyretic potential. Phytocompounds like proteins, alkaloids, flavonoids, tannins, carbohydrates, amino acids, phenolic compounds, and glycosides were already reported (Xavier et al., 2022).

To establish a solid scientific basis for the use of OEE, effective profiling of flavonoids and phenolics (the largest phytochemical molecules) with antioxidant characteristics is required (Sun and Shahrajabian, 2023). The significant levels of total phenolic and flavonoid compounds in OEE played a significant role in reducing oxidative stress via regulating various pathways, i.e., radical scavenging or inhibiting nuclear factor kappa or COX-2 enzymatic reactions.

Plants have an innate ability to biosynthesize a wide range of non-enzymatic antioxidants capable of attenuating ROS-induced oxidative damage (Kasote et al., 2015; El-Nashar et al., 2022). The possible mechanisms of antioxidant activity of OEE include the scavenging of free radicals, probably through hydrogen-holding capacity and oxidation by peroxy radicals. The results of the DPPH and ABTS assays showed radical scavenging capability (Sujarwo and Keim, 2019), while the FRAP assay helped stabilize the unstable free radicals by donating electrons to them. The antioxidant capacity of OEE indicated the presence of phenolic and flavonoid compounds with several hydroxyl groups (Lekouaghet et al., 2020) that exhibited strong antioxidant activities.

Most of the potential health benefits of polyphenols reported by HPLC analysis arise from their antioxidant activities, which are mediated by hydroxyl groups that scavenge free radicals and/or by chelate metal ions. Chlorogenic acid is a naturally occurring phenolic compound that has therapeutic or preventive effects in a variety of pathological conditions, including oxidative stress and inflammation (Bagdas et al., 2020). Gallic acid was reported to alleviate the noxious effects of inflammation and oxidative stress by improving the oxidative defense system (Moradi et al., 2021). 4-Hydroxybenzoic acid is a biodegradable, bioactive natural compound present in medicinal plants and food products, revealing the antioxidant properties that contribute to specified pharmacological properties (Mohammed et al., 2019). Caffeic acid is a natural metabolite in the plant kingdom and has antioxidant and anti-inflammatory effects due to the modulation of iNOS and other inflammatory markers (Afsar et al., 2015; Karan et al., 2021). Ferulic acid reduces the number of exuded neutrophils and is a potent inhibitor of IL-8 production, thus acting as the main component for anti-inflammatory action. Coumarin and related compounds exert a variety of pharmacological properties, such as antioxidant and anti-inflammatory effects, which can be attributed to their free radical scavenging properties (Garg et al., 2020).

Lipoxygenase enzymes transform polyunsaturated fatty acids into physiologically active markers linked to inflammatory responses and contribute to the biosynthesis of leukotrienes (Abdel-Mageed et al., 2014). Therefore, inhibition of lipoxygenase is thought to be an effective option for the treatment of a variety of inflammatory disorders. The anti-lipoxygenase efficacy of OEE revealed that it is involved in blocking the synthesis of prostaglandins and leukotrienes, which are associated with the development of inflammatory diseases (Lončarić et al., 2021). During inflammation, lysosomal enzymes are released from phagocytes into extracellular cavities, causing further damage to tissues and inducing several other disorders (Debnath et al., 2013). The result showed that OEE stabilizes the HRBC membrane from lysis and prevents the release of inflammatory mediators by lysosomal enzymes.

Acute toxicity studies confirmed the wide safe margin for the use of OEE and demonstrated its low toxicity profile (Khalid et al., 2023). Carrageenan is a chemical that increases the local blood flow and capillary permeability, resulting in edema formation. This may lead to leukocyte migration and cause inflammation (Mehrzadi et al., 2021). In this study, OEE produced an anti-inflammatory effect against carrageenan-induced edema in animal models, which might be due to the presence of polyphenols.

The analgesic potential of OEE was evaluated using the hot plate test, tail flick assay, formalin-induced pain test, and acetic acid-induced writhing test. The results showed that OEE displays a significant pain-relieving effect (analgesic) in both the tail flick and hot plate assays, which are helpful in demonstrating centrally mediated antinociceptive responses (Fan et al., 2014). Formalin-induced pain is a valid and reliable model for acute inflammation, while the acetic acid-induced writhing assay depicts peripheral analgesic properties. The analgesic activity of OEE may be due to its ability to reduce PGE2 synthesis and the levels of the inflammatory cytokines TNF-alpha, IL-1, and IL-6 (Gupta et al., 2015).

Yeast provokes pathogenic fever through the production and accumulation of prostaglandins in the hypothalamus (Sirohi and Sagar, 2019), increasing rectal temperature. OEE shows antipyretic activity by inhibiting cyclooxygenase and, consequently, decreasing PGE2 levels in the hypothalamic region, which is the main mechanism of action of most antipyretic drugs.

The process of molecular docking was employed to theoretically assess the interactions between ligands and enzymes, aiming to comprehend the underlying molecular mechanisms responsible for the diverse biological activities exhibited by natural products. This approach offers an enhanced understanding of the unique mechanism of action and binding affinity exhibited by active ligands when interacting with enzymes. To understand the inhibition potential, six phenolic compounds identified using the HPLC analysis (chlorogenic acid, gallic acid, 4-hydroxybenzoic acid, caffeic acid, ferulic acid, and coumarin), along with indomethacin (standard), were docked against the lipoxygenase enzyme. The docking results showed significant binding affinities and predicted the basis of the binding energies of these compounds to the enzyme active site. The results demonstrated that higher negative values of binding energies relate to the higher affinities of the docked compound with the enzyme. Furthermore, they predicted the interactions between the amino acid residues of the lipoxygenase macromolecule and different reaction portions of ligand molecules. These interactions gave a possible mechanism for binding the phenolic compounds to the active site of the lipoxygenase macromolecule. In conclusion, molecular docking results describe the interaction of lipoxygenase with the ligands (chlorogenic acid, gallic acid, 4-hydroxybenzoic acid, caffeic acid, ferulic acid, and coumarin) characterized by HPLC analysis, confirming our findings for the plant extract in terms of lipoxygenase inhibition assay.

## 5 Conclusion

The current study determines the phytochemical profiling and antioxidant, anti-inflammatory, analgesic, and antipyretic activities of the crude ethanolic extract of *O. esculentum* (L.f.) Sm. The HPLC analysis revealed the presence of chlorogenic acid, gallic acid, 4-hydroxybenzoic acid, ferulic acid, caffeic acid, and coumarin, which are associated with many pharmacological activities. *In vitro* and *in vivo* studies of this plant extract exhibited significant anti-inflammatory, analgesic, and antipyretic activities in a dose-dependent manner. The high binding energy of these phytocompounds with the lipoxygenase enzyme through the molecular docking study validated the anti-inflammatory potential of *O. esculentum*. Thus, the current study confirmed the use of this plant in the treatment of pain and inflammatory disorders, offering the pharmacological basis for its traditional use.

## Data Availability

The original contributions presented in the study are included in the article/Supplementary Material; further inquiries can be directed to the corresponding author.
